# Physiological and molecular dynamic changes during 23-day high-altitude exposure reveal novel biomarkers for acclimatization

**DOI:** 10.3389/fphys.2026.1763837

**Published:** 2026-02-18

**Authors:** Ling Chen, Xuefei He, Hao Wang, Sihu Hu, Haitong Zhao, Guohua Ni, Hui Yan, Lei Chen, Cheng Deng, Fengming Luo

**Affiliations:** 1 Department of High Altitude Medicine, High Altitude Medicine Key Laboratory of Sichuan Province, Center for High Altitude Medicine, West China Hospital, Sichuan University, Chengdu, Sichuan, China; 2 Laboratory of Pulmonary Immunology and Inflammation, Frontiers Science Center for Disease-related Molecular Network, West China Hospital, Sichuan University, Chengdu, Sichuan, China; 3 Institute of High Altitude Medicine, National Clinical Research Center for Geriatrics, West China Hospital, Sichuan University, Chengdu, Sichuan, China; 4 Department of Respiratory and Critical Care Medicine, West China Hospital, Sichuan University, Chengdu, Sichuan, China; 5 Emergency Management Office, West China Hospital, Sichuan University, Chengdu, China; 6 Center for High Altitude Medicine, Xining, Qinghai, China

**Keywords:** acclimatization process, acute mountain sickness (AMS), gene expression pattern (GEP), high-altitude exposure, temporal transcriptome analysis

## Abstract

**Introduction:**

Health conditions associated with rapid ascent to high altitudes remain prevalent and pose an ongoing challenge. While acute mountain sickness (AMS) typically occurs within the first few days after ascent, the physiological and molecular acclimatization processes during prolonged high-altitude exposure beyond the initial acute phase remain incompletely understood.

**Methods:**

This cross-sectional study investigated physiological and transcriptomic dynamics during prolonged high-altitude exposure over a 23 day period at 4,104 m in 113 Chinese Han individuals. Linear regression analysis, time series analysis, enrichment analysis, and protein-protein interaction analysis were applied to reveal the physiological and molecular dynamic changes.

**Results:**

Four physiological parameters (saturation of peripheral oxygen [SpO_2_], hemoglobin, hematocrit, and standard deviation of red blood cell distribution width [RDW-SD]) exhibit a significant positive linear trend with the duration of acclimatization at high altitude (DAHA). Notably, two distinct gene expression patterns (GEPs) following DAHA were characterized for the first time: a decreasing expression pattern (Pattern 1) and a “mountain-shaped” expression pattern—upregulated in the first week and then downregulated (Pattern 2). In comparing individuals who experienced or were experiencing acute mountain sickness (eAMS+, n = 56) with those who did not (eAMS-, n = 57), RNA-seq performed in a subset of 48 participants (eAMS+, n = 35; eAMS-, n = 13) identified 583 upregulated and 104 downregulated genes in the eAMS+ group. Among these, 398 upregulated genes and 10 enriched pathways were found to overlap with Pattern 2. By integrating baseline data from the GSE75665 database, five hub differentially expressed genes (DEGs)—*BCL2L1*, *DCAF12*, *CDC34*, *PINK1*, and *UBB*—were identified. These genes not only predict AMS susceptibility but also associated with molecular responses to prolonged high-altitude exposure. In particular, *CDC34* and *UBB* are novel genes not previously mentioned.

**Conclusion:**

This study provides critical insights into key physiological trends and molecular expression dynamics associated with prolonged exposure to high-altitude environments.

## 1 Introduction

Rapid ascent to high altitude induces a series of hypoxia-driven pathophysiological responses that collectively aim to maintain tissue oxygen homeostasis. These responses involve oxygen content restoration and compensatory oxygen delivery mechanisms (Julian, 2007; Zhang et al., 2014; Calbet et al., 2002), as reflected in the hypoxic ventilatory response (Weil et al., 1970; Swenson et al., 1985), hypoxic pulmonary vasoconstriction (Dehnert et al., 2005; Swenson, 2013), and changes in cerebral blood flow (Lucas et al., 2011). Together, these processes constitute the physiological basis of human acclimatization to hypobaric hypoxia following ascent to high altitude, representing an environmentally driven and non-genetic state distinct from long-term genetic adaptation (Cheng et al., 2025).

Despite the existence of shared acclimated mechanisms, individual responses to hypoxia vary substantially. Such inter-individual heterogeneity is closely linked to susceptibility to acute mountain sickness (AMS), a common high-altitude that typically manifests within the first few days after ascent (Sikri and Bhattachar, 2017).

AMS remains high prevalent, particularly among the lowland Han population (Buroker et al., 2012), and poses considerable economic burdens, safety issues (Hultgren, 1979), and even life-threatening situations (Luks et al., 2017). Previous researches have shown that physiological, cellular, and molecular responses occurring shortly after altitude exposure are crucial to AMS onset and development (Basnyat and Starling, 2015). However, the biological mechanisms underlying differential AMS susceptibility among individuals exposed to similar hypoxic environments remain incompletely clear.

Advances in genomic and transcriptomic profiling have enabled the identification of genetic variants and molecular pathways associated with hypoxia acclimatization and AMS susceptibility (Sharma et al., 2022). Several transcriptomic studies have specially investigated AMS, reporting differential expression of genes involved in inflammation, immune regulation, oxidative stress, and energy metabolism during the early phase of high-altitude exposure. Existing studies have implicated pathways related to inflammatory and immune response (Pham et al., 2022), cellular metabolism (Chen et al., 2012), and erythropoiesis (Pham et al., 2024) in hypoxia responses and altitude-related physiological adjustment (Subudhi et al., 2014). Nevertheless, most prior transcriptomic investigations have focused on early or acute exposure periods, and the relationships among gene expression patterns, biological pathways, and physiological phenotypes during prolonged high-altitude exposure beyond the initial acute phase remain poorly characterized. In particular, it is unclear whether distinct temporal gene expression patterns emerge at the population level and how such patterns relate to AMS susceptibility and physiological adjustment.

To address these gaps, we aimed to gain a more comprehensive understanding of the acclimatization process by analyzing dynamic physiological and molecular changes following high-altitude exposure. Physical examination, AMS symptoms assessment and RNA sequencing (Liew et al., 2006; Kohane and Valtchinov, 2012) were performed. A time-point-based analytical framework was applied across individuals with varying durations of high-altitude exposure to evaluate exposure-associated dynamic under hypobaric hypoxia. Gene clustering, enriched pathway analysis, and network analysis were used to identify gene expression patterns (GEPs) and hub genes for AMS susceptibility and acclimatization. In total, 113 Chinese Han individuals exposure to high altitude for up to 23 days were enrolled. Four physical parameters exhibited a positive linear relationship with the duration of acclimatization at high altitude (DAHA). Two distinct GEPs and their associated biological pathways were identified. In addition, five hub genes with two novel genes were found as potential key biomarkers for AMS susceptibility and the acclimatization process.

## 2 Results

### 2.1 Demographic characteristics

A total of 113 Chinese Han individuals (104 males, 9 females), aged between 18 and 63, were enrolled in this cross-sectional study. Among them, 56 had experienced or were experiencing acute mountain sickness (eAMS+), while 57 did not. DAHA ranged from 1 to 23 days (Supplementary Figure S1; Table 1). Participants were categorized into four groups based on DAHA: T1 (≤3 days, n = 22), T2 (4–7 days, n = 18), T3 (8–14 days, n = 29), and T4 (15–23 days, n = 44). The Kruskal–Wallis test revealed no significant demographic differences (sex, age, BMI, eAMS) among the four DAHA groups (Table 2). Chi-square test or t-test suggested no demographic differences among eAMS + group and eAMS- group (Table 3).

**TABLE 1:** Distribution of population characteristics.

Characteristics	Min	1st Qu	Median	Mean	3rd Qu	Max
Age(y/o)	18	29.00	37.00	38.39	47.00	63.00
BMI(kg/m^2^)	16.60	21.70	23.40	23.51	25.10	33.80
eAMS score	0	0	0	1.91	4.00	8.00
DAHA(days)	1.00	5.00	13.00	10.96	15.00	23.00

Qu, quartile; BMI, body mass index; eAMS, experienced or experiencing acute mountain sickness; DAHA, days of acclimatization at high altitude.

**TABLE 2:** Bias results of population characteristics among the four DAHA groups.

Characteristics	χ^2^	df	p value
Gender	2.63	1	0.1051
Age(y/o)	36.98	35	0.3778
BMI(kg/m^2^)	60.17	63	0.5778
eAMS(+/−)	6.93	5	0.2262

χ^2^, chi-square, df, degrees of freedom.

**TABLE 3:** Bias results of population characteristics among eAMS+ and eAMS- groups.

Characteristics	Level	eAMS- (n = 57)	eAMS+ (n = 56)	p value
Gender	Female	3 (5.3)	6 (10.7)	0.47
	Male	54 (94.7)	50 (89.3)	
Age (y/o)		39.65 (11.47)	37.11 (10.68)	0.226
BMI (kg/m^2^)		23.83 (3.05)	23.19 (3.16)	0.277
DAHA		11.75 (5.44)	10.16 (6.34)	0.154

BMI, body mass index; DAHA, days of acclimatization at high altitude.

### 2.2 Correlations between physiological parameters and the DAHA

**FIGURE 1:**
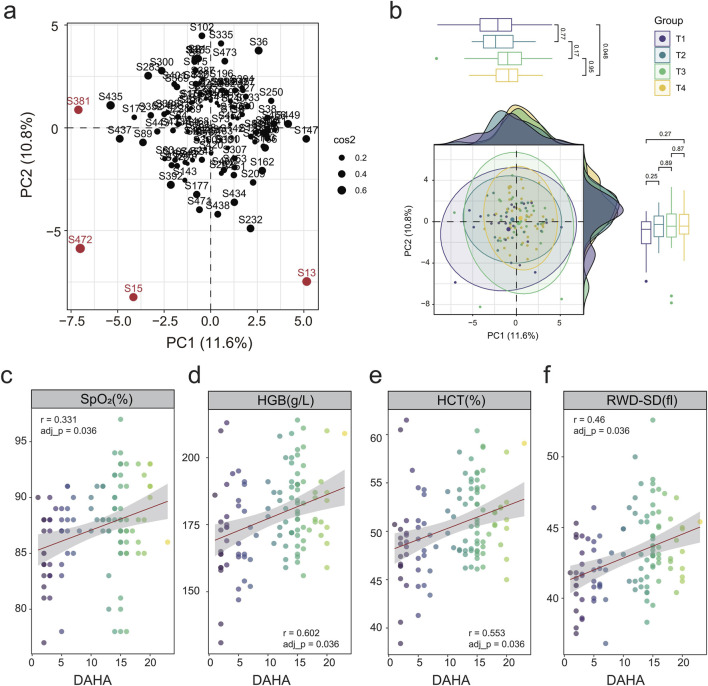
Quality control and linear regression analysis of detection parameters correlated with DAHA. **(a)** PCA plot of outliers based on the detection parameters. Red points indicate outlier samples. **(b)** PCA plots results and basic statistics between DAHA groups based on the detection parameters. Samples and circles represent the 95% confidence intervals colored according to DAHA groups. Different circles represent the 95% confidence intervals for each DAHA group c–f. Dot plots showing linear regression analyzes between DAHA and the following parameters: SpO_2_ **(c)**, HGB **(d)**, HCT **(e)**, and RWD-SD. **(f)** The points are colored according to their DAHA value. r: correlation coefficient, adj_p: adjusted p values by Benjamini & Hochberg method.

We analyzed 44 physiological parameters (Supplementary Table S1) to define acclimatization traits. After excluding sample S187 for excessive missing data (17 out of 44 items, 38.64%), principal component analysis (PCA) was performed on 112 individuals. Four additional outliers (S381, S472, S15, S13) out of 95% confidence intervals were removed (Figures 1a,b). Multiple linear regression model (adjusted for age, sex, and BMI) identified four parameters exhibiting a significant linear relationship with DAHA after adjusting for multiple comparisons via the Benjamini–Hochberg method (FDR <0.05). These parameters included saturation of peripheral oxygen (SpO_2_, r = 0.331), hemoglobin (HGB, r = 0.602), hematocrit (HCT, r = 0.553), and the standard deviation of red blood cell distribution width (RDW-SD, r = 0.46) (Figures 1c–f; Supplementary Table S2). However, only RDW-SD remained significant in the subsequent Kruskal‒Wallis test (FDR = 0.0066, Supplementary Table S3).

### 2.3 Time series analysis reveals the expression trends of genes in the DAHA groups

**FIGURE 2:**
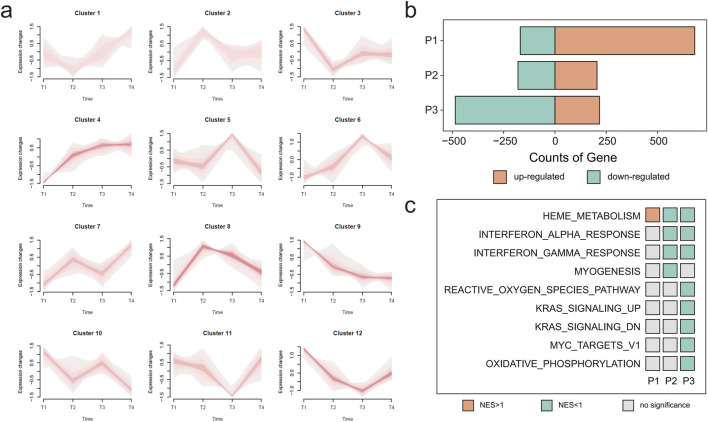
The results of the time series analysis, DEGs and HGSEA among DAHA groups. **(a)** Line plot presenting the results of the time series analysis, where genes were divided into 12 clusters. Red lines represent the dominant expression trend within each cluster across DAHA groups. **(b)** Bar chart illustrating the counts of DEGs in 3 periods (P1, P2 and P3). Orang bars: upregulated genes, Turquoise bars: downregulated genes. **(c)** Heatmap displaying the HGSEA results of 3 periods (P1, P2 and P3). Orange tiles: upregulated pathway (NES >1), Turquoise tiles: downregulated pathway (NES <1), Gray tiles: no significance pathway. Note: DAHA: days of acclimatization at high altitude; DEGs: differentially expressed genes; HGSEA: hallmark gene set enrichment analysis; P1: period 1 (T2 vs. T1); P2: period 1 (T3 vs. T2); P3: period 1 (T4 vs. T3); NES: normalized enrichment score.

To explore the relationship between GEPs and DAHA, we performed transcriptomics on a subset of 48 individuals with complete RNA sequencing data (including 35 eAMS+ and 13 eAMS- participants). Their DAHA ranged from 2 to 19 days and were divided into T1 (≤3 days, n = 4), T2 (4–7 days, n = 11), T3 (8–14 days, n = 17), and T4 (15–19 days, n = 16) (Supplementary Figures S2a,b). Using Fuzzy C-Means Clustering (FCM) clustering algorithm, genes were grouped into 12 clusters with distinct temporal dynamics. Among these, Cluster 4 (C4, n = 1,389) and Cluster 9 (C9, n = 1,469) showed consistently increasing and decreasing gene expression over time, respectively (Figure 2a; Supplementary Figure S2c). C4 was enriched in adaptive immune response, nonsense-mediated decay independent of the exon junction complex, steroid biosynthesis, and the regulation of protein transport. And C9 was linked to the regulation of the response to biotic stimuli, the innate immune response, neutrophil degradation, interferon gamma signaling, the regulation of immune effector processes, and other immune or inflammatory pathways (Supplementary Figures S2d,e).

Moreover, differentially expressed gene (DEG) analysis across DAHA groups revealed distinct gene changes in each period: P1 (T2 vs*.* T1, 681 up, 169 down), P2 (T3 vs*.* T2, 205 up, 108 down), and P3 (T4 vs*.* T3, 217 up, 486 down) (*p <* 0.05, |logFC| > 0.3785) (Figure 2b). Hallmark Gene Set Enrichment Analysis (HGSEA) revealed that heme metabolism was upregulated in P1 then downregulated in P2 and P3, aligned with, the “acute increase followed by decrease” expression trend of Cluster 8 (C8). Interferon alpha/gamma response were not different in P1 but significantly downregulated in P2 and P3 (*p <* 0.05*, q* < 0.25, and *|*Normalized Enrichment Score (NES)*|* > 1) (Figure 2c). C8 was specially enriched in heme metabolism (*p* = 1e-10, *q* = 4e-9, NES = 2.13), while the interferon alpha/gamma response was enriched in C9. In contrast, no significant HGSEA results were identified for C4 (Supplementary Table S4). Based on the expression trends and enrichment results, the functional characteristics of C4, C8, and C9 were further compared.

### 2.4 Weighted gene coexpression network analysis (WGCNA) reveals the relationships between gene modules and phenotypes

We performed WGCNA on the transcriptomic data to investigate gene modules associated with DAHA. After excluding 3 outlier samples identified by hierarchical clustering identified, a soft threshold power of 6 was selected for network construction (Supplementary Figures S3a,b). The gene set was divided into modules using an adjacency matrix, which quantifies the pairwise correlations with genes. To ensure robust module definitions, modules with over 75% similarity were merged, resulting in 26 distinct modules (Supplementary Figure S3c). Among these, the darkorange module (DO) presented a significant negative correlation with DAHA (*r* = −0.3*, p* = 0.048). Conversely, the darkmagenta module (DM) were positively correlated (*r* = 0.39*, p* = 0.0086). Significant correlations with eAMS were identified: positive for the blue module (BL; r = 0.35, p = 0.019) and negative for DO (r = −0.34, p = 0.021) (Supplementary Figure S3d).

Enrichment analysis showed that DO-associated genes (n = 114) were linked to several key immune processes such as the antiviral defense response and interferon response, sharing similar expression trends and functions with C9. In contrast, the DM (n = 37) were involved in memory and ion transport, while BL (n = 1911) was significantly enriched in protein catabolic, cellular stress responses, metabolic processes, and autophagy regulation (Supplementary Figure S4).

### 2.5 Gene expression patterns and hub genes identified over time

**FIGURE 3:**
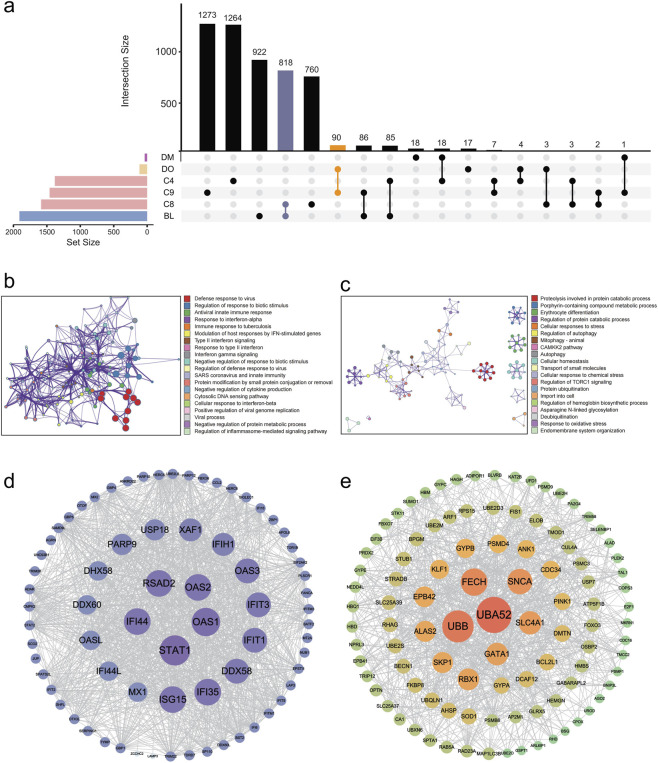
Gene counts, functional analysis, and hub genes associated with Pattern 1 and Pattern 2. **(a)** UpsetR plot of all the gene sets of interest and their intersection sets. **(b)** Enrichment analysis of the intersection genes between DM and C9 (Pattern 1). **(c)** Enrichment analysis of the intersecting genes between BL and C8 (Pattern 2). **(d)** Concentric circle plot of genes in Pattern 1. Circle size and color, as well as label size, are positively correlated with gene degree. **(e)** Concentric circle plot of the top 100 genes in Pattern 2. Circle size and color, as well as label size, are positively correlated with gene degree. Note: DM, darkmagenta module; DO, darkorange module; BL, blue module; C4, Cluster 4; C8, Cluster 8; C9, Cluster 9.

Using integrated analysis of time series and WGCNA, we identified 18 high-confidence genes shared between the DM module and C4, both positively associated with the DAHA (Figure 3a). These genes were associated primarily with the negative regulation of hydrolase activity (3 genes), inorganic ion transmembrane transport (4 genes), and intracellular chemical homeostasis (3 genes) (Supplementary Figure S5a). Although 15 of these genes were found in the STRING database, protein‒protein interaction (PPI) network analysis revealed no significant associations among them (Supplementary Figure S5b).

The gene set with a decreasing expression trend with DAHA, the DO module and C9 shared 90 immunity-related genes involved in antiviral defense, interferon responses, and tuberculosis immunity. To pinpoint key genes, we imported the PPI results into Cytoscape. The top 5% (n = 5) of genes with the highest degree centrality were selected as hub genes: *STAT1*, *OAS1*, *OAS2*, *RSAD2*, and *IFI44*. Considering the consistent expression trend and functional profile of this intersection gene set, we designated it as Pattern 1 (Figures 3a,b,d).

The BL module shared 85 genes with C4, enriched in pathways including the Nop56p-associated pre-rRNA complex (4 genes), insulin receptor signaling pathway (3 genes), cholesterol metabolic process (3 genes), positive regulation of the MAPK cascade (5 genes) and positive regulation of translation (3 genes) (Supplementary Figure S5c). It also shared 86 genes with C9, which were linked to organic acid catabolic processes (4 genes), proteinogenic amino acid metabolic processes (3 genes), and herpes simplex virus 1 infection (3 genes) (Supplementary Figure S5d).

For C8, the BL module presented the highest gene overlap. These genes were enriched in processes like protein ubiquitination, erythrocyte differentiation, autophagy, and mitophagy. The top 20 hub genes were identified via PPI network analysis. These genes comprised *UBA52*, *UBB*, *FECH*, *SNCA*, *SLC4A1*, *GATA1*, *RBX1*, *ALAS2*, *SKP1*, *EPB42*, *GYPB*, *KLF1*, *PSMD4*, *ANK1*, *CDC34*, *PINK1*, *BCL2L1*, *DMTN*, *DCAF12*, and *GYPA*. The “mountain-shaped” expression pattern of these 818 genes was defined as Pattern 2 (Figures 3a,c,e).

### 2.6 Hub genes highly correlated with the physiological phenotype

Furthermore, we investigated the correlations between the hub genes and the 44 physiological parameters mentioned above. Hub genes in Pattern 1 were negatively correlated with plateletcrit (PCT) and platelets (PLTs). Key correlations included *STAT1* negative with the percentage of monocytes (MONOP) and mean corpuscular hemoglobin concentration (MCHC). *OAS1* positive with creatinine (CREA) and aspartate aminotransferase (AST), *OAS2* positive with HCT and HGB, and *IFI44* weakly positive with the white blood cell (WBC) count.

In Pattern 2, nearly all hub genes correlated negatively with immune cells like WBC, monocyte count (MONO), MONOP, neutrophil count (NEUT), lymphocyte count (LYMPH) and hematological parameters like HCT, red blood cell count (RBC) and HGB, but were positively correlated with CREA. *UBA52*, the hub gene with the highest degree in Pattern 2, exhibited positive correlation with the percentage of eosinophils (EOP), aspartate aminotransferase/alanine aminotransferase (ASL/ALT), and direct bilirubin (DBIL), and negative correlation with the platelet distribution width (PDW), low-density lipoprotein cholesterol (LDLC), and total cholesterol (TC). Notably, *PSMD4* presented the strongest positive correlation with CREA among all Pattern 2 hub genes (Supplementary Figure S6).

### 2.7 Differences between the eAMS+ and eAMS- groups in pattern 1 and pattern 2

WGCNA revealed distinct gene network expression in DO and BL between the eAMS+ and eAMS- groups (Supplementary Figure S3d). Using a general linear model with AMS experience as the independent variable and DAHA, age, sex and BMI as the covariates, 583 upregulated genes and 104 downregulated genes were identified (Supplementary Table S5). Enrichment analysis showed that the upregulated genes were involved in adaptive immune response activity, erythrocyte differentiation and the CAMKK2 pathway (Supplementary Figure S7a). Conversely, the downregulated genes were linked to leukocyte-mediated cytotoxicity and antibacterial humoral response (Supplementary Figure S7b).

**FIGURE 4:**
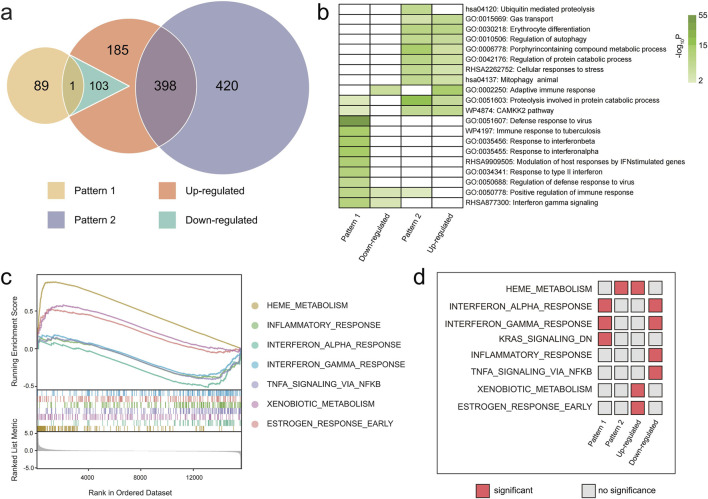
Gene set counts, overlaps, and enrichment analyzes of DEGs and GEPs. **(a)** Venn diagram showing the overlap between the eAMS + group, the eAMS- group, Pattern 1 and Pattern 2. **(b)** Heatmap showing the enrichment analysis results for the upregulated and downregulated genes between the eAMS+ and eAMS- groups, as well as for the genes in Pattern 1 and Pattern 2. Green tiles indicate the significant pathways and the depth of color represents the -logP value. **(c)** HGSEA curve plot presenting the significant hallmark genesets pathways comparing the eAMS+ and eAMS- groups. **(d)** Heatmap displaying the HGSEA results for the upregulated and downregulated genes between the eAMS+ and eAMS- groups, as well as for the genes in Pattern 1 and Pattern 2. The red box indicates significant results, and the gray box suggests no significance.

We identified 398 upregulated genes and 10 pathways shared with Pattern 2, such as gas transport, erythrocyte differentiation, autophagy regulation and so on. In addition, only one downregulated gene overlapped with Pattern 1, though both showed enrichment in immune response and interferon gamma signaling (Figures 4a,b). The HGSEA results further revealed upregulation of heme metabolism and xenobiotic metabolism pathway, and downregulation of interferon response and inflammatory pathways in the eAMS + group (Figure 4c). Heme metabolism was also found in Pattern 2, while the interferon responses were common in Pattern 1 (Figure 4d). These findings highlight molecular distinctions between eAMS+ and eAMS- groups and their strong association with both Pattern 1 and Pattern 2.

### 2.8 *BCL2L1*, *DCAF12*, *CDC34*, *PINK1*, and *UBB* were identified as potential biomarkers for AMS prediction and the acclimation process

**FIGURE 5:**
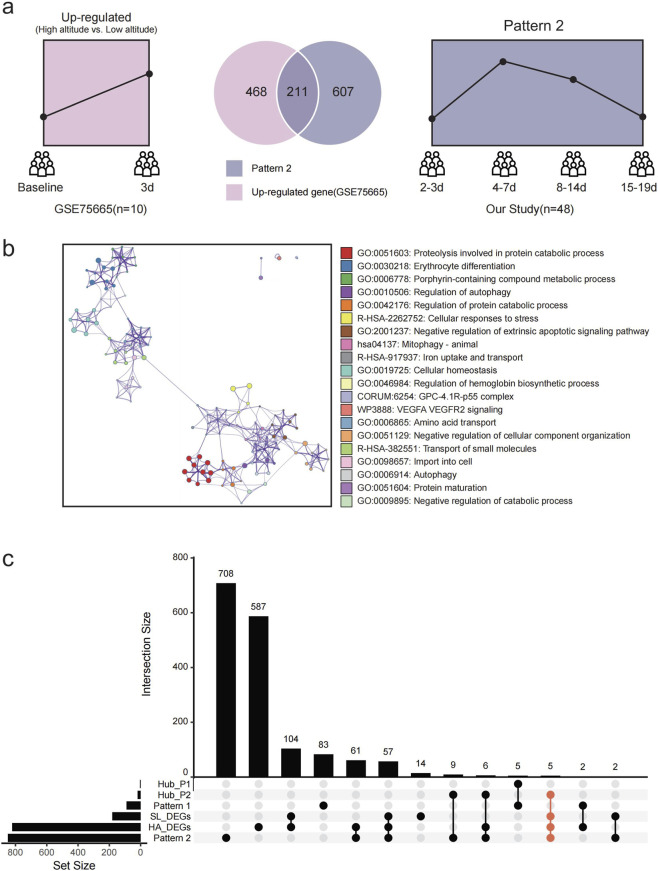
Changes in gene expression from low altitude to high altitude in Pattern 2. **(a)** Trends and counts of upregulated genes in GSE75665 and in Pattern 2 identified in our study. Pink: data from GSE75665, Darkblue: data from our study. **(b)** Enrichment analysis results for the genes shared between the upregulated genes in GSE75665 and Pattern 2 from our study. **(c)** UpSetR plot showing gene sets of Pattern 1, Pattern 2, hub genes in Patterns 1 and 2, DEGs from sea level and high altitude in dataset GSE75665, and their overlaps.

To contextualize our findings, we integrated our Pattern 2 data with the public dataset GSE75665, which tracks transcriptomic changes in human blood before and after 3-day high-altitude exposure. After high-altitude exposure, 679 genes were upregulated and 1,020 downregulated compared with their baseline conditions. Of these, 211 upregulated genes overlapped with Pattern 2 in our study. Enrichment analysis revealed that the gene set shared was primarily associated with proteolysis, erythrocyte differentiation, porphyrin metabolism, and the regulation of autophagy and protein catabolism (Figures 5a,b).

At low altitudes, 64 of the 182 DEGs between AMS and non-AMS groups overlapped with Pattern 2. These included five Pattern 2 hub genes (*BCL2L1, DCAF12, CDC34, PINK1 and UBB*), highlighting their potential as predictive biomarkers for AMS (Figure 5c). After high-altitude exposure, the overlapped genes with Pattern 2 increased to 128 DEGs with eleven hub genes (*ALAS2, BCL2L1, DCAF12, CDC34, EPB42, GATA1, PINK1, PSMD4, SNCA, UBA52, and UBB*) including the five initial biomarkers (Figure 5d). Conversely, in the GSE75665 data, Pattern 1 was nearly absent, found in none of the 182 low-altitude DEGs and only 2 high-altitude DEGs (*EIF2AK2* and *TRIM22*) (Figure 5c; Supplementary Tables S6,S7).

## 3 Discussion

In our research, the phenotypic responses and molecular expression patterns were examined and analyzed among the Chinese Han individuals following exposure to an altitude of 4,014 m over a period of 23 days. The biological response to prolonged high-altitude exposure is complex and temporally dependent (Figure 2a). SpO_2_, HGB, HCT, and RDW-SD exhibited positive linear relationships with DAHA. Using a combination of transcriptomics analysis and WGCNA, a decreasing expression pattern (Pattern 1) and a “mountain-shaped” expression pattern (Pattern 2) were identified. Pattern 1 was associated with immunity-related pathways, and Pattern 2 was associated with protein ubiquitination and catabolic processes, erythrocyte differentiation, cellular autophagy, and mitophagy. Nearly all the hub genes in Pattern 2 correlated with the physiological parameters, indicating that Pattern 2 could be involved primarily in the process of acclimatization to high altitudes. Compared with those in the eAMS- group, 583 upregulated DEGs and 104 downregulated DEGs were found in the eAMS + group. Among them, 398 upregulated genes and 10 pathways were associated with Pattern 2, whereas one downregulated gene and two pathways overlapped with Pattern 1, indicating that the molecular differences between the eAMS+ and eAMS- groups were associated with both Pattern 1 and Pattern 2. By integrating low-altitude baseline data from the GSE75665 database, five DEGs before and after altitude exposure that matched the hub genes of Pattern 2 were identified. These findings suggested that the five hub genes could serve as potentially predictive biomarkers for AMS and may be differentially involved in the high-altitude acclimatization process between the eAMS+ and eAMS- groups.

Our study revealed that the SpO_2_, HGB, HCT, and RDW-SD levels gradually increased over time (Figure 1c). The increasing trend of SpO_2_ and pulse rate (P) after exposure to high altitude was consistent with the findings of previous studies (Gaur et al., 2020). Milledge and Cotes (1985) observed that serum erythropoietin levels increased one and 2 days after reaching an altitude of 3,500 m and then returned to baseline values even when the elevation increased to 4,500 m. However, the HCT level continued to rise steadily, as evidenced by the gradual increase in HGB and HCT levels in our findings. RDW-SD, which reflects changes in the size of erythrocytes, also gradually increased, altogether suggesting that erythrocytes underwent various degrees of modifications to enhance their oxygen delivery capacity. Almost all the hub genes of Pattern 2 were negatively correlated with HCT, RBC, WBC, and HGB. These findings suggest that the hub genes in Pattern 2 are involved mainly in pathways related to heme metabolism and erythropoiesis. The physiological parameters confirmed this result. The lower the parameters, such as hemoglobin, the higher the gene expression of this pattern. Acute systemic hypoxia and long-term renal hypoperfusion may cause creatinine to increase (Zhou et al., 2013), however, the direct correlation between creatinine and heme metabolism is unclear. These findings suggest that erythrocyte-related indicators accompanied by heme metabolism and erythropoiesis are important hypoxic response to prolonged high-altitude exposure.

Although some studies have explored molecular changes (Yang et al., 2024; Pham et al., 2024) within the first 3 day high-altitude exposure are associated with the pathogenesis of AMS, the patterns of gene expression following prolonged exposure exceeding 3 days are rarely investigated. Previous studies have shown that upregulated pathways include those involved in blood gas transport, increased erythrocyte development and differentiation, and the heme biosynthetic process during the first 3 day high-altitude exposure (Zhao et al., 2024). Priya Gaur and her colleagues (Gaur et al., 2020) explored physiological parameters and differentially expressed genes at high altitudes on days 3, 7, 14, and 21. In this study, day 3 at high altitude was found to be a distinct time point. However, none of the sea-level volunteers experienced or were experiencing AMS, and the gene expression patterns were not analyzed. In our study, we analyzed the temporal gene expression dynamics of individuals and identified the “mountain-shaped” GEP (Pattern 2) for the first time. This pattern indicated that the peak occurred at T2, which was 3–7 days after ascending to high altitude. This finding suggested that although clinical symptoms visibly alleviate within 7 days, molecular expression remains increasing. The transcripts in Pattern 2 are associated primarily with these pathways, e.g. erythrocyte differentiation, proteolysis, autophagy, mitophagy, gas transport, and CAMKK2, some of which have been mentioned in previous studies (Zhao et al., 2024). These results indicated that these biological processes persisted at least until the 7th day after acute ascent to high altitude.

The stability of redox status (Paul et al., 2018), immune and inflammatory responses (Pham et al., 2024; Song et al., 2016), and erythropoiesis (Pham et al., 2024; Tomar et al., 2015) are important pathways during the acute altitude acclimatization process. It remains unclear what differences in bioinformatics pathways are between individuals with and without AMS. Our study revealed that the eAMS + group presented a transcriptional profile indicative of an enhanced yet potentially dysregulated adaptive response. Specifically, “enhanced” acclimatization in this context refers to the broad activation of immune and hypoxia-responsive pathways, reflecting an intensified systemic effect to cope with hypoxic stress. In contrast, “dysregulated” acclimatization is suggested by the coexistence of immune activation with impaired downstream effector functions. Although pathways related to adaptive immunity are significantly upregulated in eAMS + individuals, the concurrent downregulation of leukocyte-mediated cytotoxicity and neutrophil degranulation suggests a disconnect immune signaling and effective immune execution. Such an imbalance may result in insufficient pathogen clearance or prolonged, unresolved inflammation, thereby contributing to AMS pathophysiology. Conversely, the increase in erythrocyte differentiation and gas transport represents a canonical compensatory effort to enhance oxygen delivery (Golden et al., 1981) in response to hypoxic stress. However, the significant enrichment of hydrogen peroxide catabolic processes points to elevated oxidative stress (Rojkind et al., 2002), which may exacerbate cellular damage. The increased activity of CAMKK2, a key hypoxia-sensitive pathway (Li C. et al., 2023), further supports a heightened metabolic stress response in eAMS + individuals. These three pathways, including heme metabolism, overlapping with Pattern 2, the interferon alpha response, and the interferon gamma response overlapping with Pattern 1, were more significant in eAMS + individuals than in eAMS- individuals (Figure 4d). These findings indicate that eAMS + individuals experience more pronounced physiological disruption and immune dysregulation. Overall, the eAMS + group presented greater metabolic stress and more significant immune suppression, highlighting key mechanisms underlying its poorer acclimatization to high-altitude environments.

Molecular biomarkers are crucial in high-altitude pathophysiological conditions. One study identified candidate genes controlling cellular interactive pathways suggesting that hypoxia affects the structure and function of endothelial cells (Pham et al., 2024). Another study highlighted ten key genes involved in blood gas transport, erythrocyte development and differentiation, and heme biosynthetic process. Two of these key genes, FECH and ALAS2, play crucial roles in the heme biosynthetic pathway (Norboo et al., 2004), which was also detected in our hub 20 genes of Pattern 2 (Figure 3e). Through joint analysis with the GSE75665 database in our study, five predictive hub genes (*BCL2L1*, *DCAF12*, *CDC34*, *PINK1* and *UBB*) were identified and were significantly differentially expressed between the AMS group and the non-AMS group before and after high altitude exposure. These five hub genes form a synergistic network: *BCL2L1* promotes cellular survival (Chen et al., 2015), *DCAF12* (Jiao et al., 2022; Patron et al., 2019) and *CDC34* (Ben-Neriah, 2002) enable targeted degradation, regulating specific stress responses, inflammation, and the cell cycle; *PINK1* regulates hypoxia-related mitochondrial function, and *UBB* underpins essential proteostasis. The coordinated expression of these genes reflects the cellular struggle to maintain homeostasis under hypoxicstress, making them candidates for a multi-gene AMS prediction panel. These five genes serve as crucial molecules within the intricate biological processes of apoptosis and ubiquitination which play vital roles in the hypoxic environment. *ALAS2*, *BCL2L1* and *DCAF12* have been found to be significantly expressed 10 days after arriving at altitude, and *BCL2L1* had the highest sensitivity and specificity following application of the adaptive model (Wang et al., 2017). Controlling the mitochondria quality by promoting PINK1/parkin-dependent mitophagy and mitochondrial fission could alleviates AMS (Tian et al., 2023; Li Y. P. et al., 2023). Interestingly, *CDC34* and *UBB* are rarely mentioned in previous hypoxic or high-altitude studies. Further validation through clinical investigations and experimental studies is essential to establish their predictive value. This could ultimately contribute to improved strategies for the prevention and management of AMS in vulnerable Han populations, such as workers and travelers in high-altitude environments (Norboo et al., 2004; Fiore et al., 2010).

## 4 Limitations

Several limitations should be considered when interpreting our findings. Firstly, due to cross-sectional design, physiological and transcriptomic profiles obtained from different individuals at varying DAHA enables the identification of population-level exposure-associated trends, but they do not permit direct inference of within-individual longitudinal dynamics. Accordingly, the two gene expression patterns identified in this study should be interpreted as exposure duration-associated expression profiles across the cohort rather than true dynamic trajectories within the same individual. Additionally, AMS symptoms within the first 3 days after ascent were assessed using a standardized questionnaire, and for some participants were recorded retrospectively based on recall, which may introduce recall bias despite the short recall window. Moreover, owing to the lack of baseline data before exposure to high altitude, this study conducted a comprehensive analysis by integrating the baseline data from the GSE75665 database, which may introduce residual variability.

Finally, while *CDC34* and *UBB* were identified as novel hub genes associated with Pattern 2 and AMS susceptibility, their functional role in high-altitude acclimatization remains to be experimentally validated. Future studies employing hypoxia-treated cell culture models (such as endothelial cells, immune cells, or erythroid progenitors) as well as *in vivo* hypoxia or high-altitude animal models could be used to investigate the mechanistic involvement of *CDC34* and *UBB* mediated pathways in hypoxic acclimatization, inflammatory regulation, and cellular stress responses. Such longitudinal and experimental approaches will be essential to confirm causality and to further elucidate the molecular mechanisms underlying high-altitude acclimatization.

## 5 Conclusion

Our research provides significant insights into the gene expression patterns associated with the process of prolonged high-altitude exposure. Furthermore, physiological and molecular responses were significantly different between individuals with and without eAMS during the acclimatization process. The hub genes were not only potential predictive biomarkers for AMS but also key biomarkers participating in the acclimatization process.

## 6 Research design and methods

### 6.1 Study design

The study was designed as a cross-sectional investigation of physiological and molecular characteristics associated with high-altitude exposure. A total of 113 Chinese Han individuals who recently arrived at a high altitude (LiTang, 4,104 m) were enrolled between February 29th and 6 March 2024. And those with diabetes, respiratory disease, cardiovascular or neurological diseases were excluded. All participants underwent a single standardized physical examination and blood sampling during their stay at high altitude. Meanwhile, participants completed structured case report form (CRF) questionnaires to document demographic characteristics and AMS symptoms occurring within the first 3 days after ascent. Throughout the high-altitude exposure period, they received standardized meals with comparable nutritional contents and were living and working in the same environment. The study was approved by the Ethics Committee of West China Hospital (No. 2021–716). All participants provided written informed consent for the use of their samples and data for research purposes.

### 6.2 Diagnosis of AMS

The symptoms of AMS include headache, gastrointestinal issues, fatigue and/or weakness, and dizziness/light-headedness. AMS was diagnosed via the 2018 Lake Louise Acute Mountain Sickness Score (LLAMS) (Roach et al., 2018), which identifies individuals with a headache score of at least one point and a cumulative score of at least 3 points. For participants who examined within the first 3 days after ascent, AMS symptoms were recorded prospectively. For participants who had already resided at high altitude for more than 3 days at the time of assessment, AMS symptoms occurring within the first 3 days after ascent were recorded retrospectively based on participant recall, using a structured questionnaire administered by trained investigators. Participants who experienced or were experiencing AMS during the early post-ascent period were defined as the eAMS + group, and individuals who did not meet these criteria were classified as eAMS negative (eAMS-) group, which served as the internal control group.

### 6.3 Blood collection and storage

Blood was collected from a peripheral vein after 12 h of fasting at high altitude. As this was a cross-sectional study, blood sampling was performed once at a single time point for each individual. The duration of high-altitude exposure at the time of blood collection was retrospectively determined based on each participant’s documented arrival date at high altitude, resulting in exposure durations ranging from 1 to 23 days.

Blood samples collected for complete blood cells (CBC) count and blood biochemical tests were immediately sent to LiTang County People’s Hospital for testing. A subset of 48 participants, selected from the overall cohort, provided samples for RNA sequencing. All blood samples of the RNA-sequencing set were stored at −80 °C with PAXgene Blood RNA Tubes (PAXgene, PreAnalytix, Hombrechtikon, Switzerland, distributed by Qiagen, catalog no. 762165) until subsequent RNA extraction and sequencing.

### 6.4 RNA extraction, library construction, sequencing and analysis

Total RNA was extracted from blood samples collected from a subset of 48 participants selected from the original cohort of 113 individuals, using lysis, PAXgene spin columns, and DNase treatment. After quality assessment, mRNA was enriched, globin mRNA removed, and fragmented for cDNA synthesis. Following library preparation with dUTP-based second-strand synthesis, end repair, A-tailing, and adapter ligation, DNA nanoballs (DNBs) were generated and sequenced on the BGI-T7 platform (BGI-Shenzhen, China) using combinatorial probe-anchor synthesis (cPAS) to achieve high-throughput pairwise sequencing.

The raw sequencing data were filtered via SOAPnuke (Chen et al., 2018) (version 1.6.5) to remove the following reads: 1) reads containing adapters (adapter contamination), 2) reads with an unknown base N content exceeding 1%, and 3) low-quality reads (where the proportion of bases with a quality score below 15 exceeded 40% of the total read length). Clean reads were aligned to the *Homo sapiens* reference genome (GRCh38.p14) via STAR (Dobin et al., 2013) (version 2.7.10b) and gene expression counts were quantified via featureCounts (Liao et al., 2014) (version 2.0.4). Normalization was calculated via the trimmed mean of M values (TMM) method in the R package edgeR (Robinson et al., 2010) (version 4.0.16). Differentially expressed genes (DEGs) between the eAMS+ and eAMS- groups were identified using Generalized Linear Model (GLM) (adjusted for DAHA, age, sex and BMI) with significance defined as p < 0.05 and |logFC| > 0.3785.

### 6.5 Time series expression analysis and coexpression network analysis

Based on the duration of high-altitude acclimatization (Lucas et al., 2011; Gaur et al., 2020), the subset of 48 participants for RNA sequencing analysis had exposure duration ranging from 2 to 19 days (Supplementary Figure S2a). These participants were divided into 4 time-point groups (T1: ≤3 days; T2: 4–7 days; T3: 8–14 days; T4: 15–19 days) (Supplementary Figure S2b). Time series gene expression analysis was performed using FCM algorithm from the R package Mfuzz (Kumar and E Futschik, 2007) (version 2.62.0) to categorize genes into 12 clusters on the 4 time-point groups. Furthermore, Weighted Co-Expression networks were constructed using the R package WGCNA (Langfelder and Horvath, 2008) (version 1.73) to identify the relationships among genes and investigate their associations with specific traits.

### 6.6 Enrichment and protein‒protein interaction analysis

Gene enrichment analysis was conducted via the Metascape (Zhou et al., 2019) (version 3.5) (https://metascape.org/) with default settings by inputting unique gene symbols from each gene set of interest. Hallmark Gene Set Enrichment Analysis (GSEA) (Subramanian et al., 2005) was utilized to identify significant pathways (p < 0.05, q < 0.25, |NES| > 1) between the AMS and non-AMS groups. PPI network from STRING (Szklarczyk et al., 2023) (version 12.0) (https://string-db.org/) was visualized in Cytoscape (Shannon et al., 2003) (version 3.10.3). Hub genes were defined as those in the top 2.5% or 5% of node degree based on the size of the gene set.

### 6.7 Public data collection and analysis

For compensating the lack of baseline data and prediction biomarker identification, we conducted a systematic literature search for studies associated with AMS. Inclusion criteria were: 1). The transcriptomics data is available; 2). The participants should have baseline data and AMS outcomes following high-altitude exposure; 3). As genetic and environmental heterogeneity significantly influences gene expression patterns (Martin et al., 2014), we restricted the baseline data to the Chinese population. Following our inclusion criteria, only one public transcriptome data GSE75665 (Liu et al., 2017) was included. This dataset comprised 20 samples divided into two groups (AMS and non-AMS) and two time points (baseline and 72 h after high-altitude exposure). DEGs based on longitudinal groups were analyzed via a density-based pruning algorithm. For the baseline cross-sectional comparison (AMS vs. non-AMS = 5 vs. 5), we employed the same analytical approach, excluding the lowest 10% expressed genes and considering |logFC| > 0.3785 as statistically significant.

## Data Availability

The data presented in this study are deposited in the China National Center for Bioinformation (CNCB-NGDC) repository, accession number OMIX011838.

## Glossary

DAHA: duration of acclimatization at high altitude
eAMS: the experienced or experiencing acute mountain sickness
AMS: acute mountain sickness
HGSEA: hallmark gene set enrichment analysis
WGCNA: weighted gene coexpression network analysis
GEP: gene expression pattern
DEGs: differentially expressed genes
FCM: Fuzzy C-Means
CRF: case report form
NES: normalized enrichment score
|NES|: absolute normalized enrichment score
|logFC|: absolute log fold change
PPI: protein-protein interaction
PCA: principal component analysis
BMI: body mass index
P: pulse rate
SpO2: saturation of peripheral oxygen
SBP: systolic blood pressure
DBP: diastolic blood pressure
TBIL: total bilirubin
DBIL: direct bilirubin
IBIL: indirect bilirubin
TP: total protein
ALB: albumin
GLB: globulin
AST: aspartate aminotransferase
ALT: alanine aminotransferase
CREA: creatinine
UA: uric acid
BUN: blood urea nitrogen
TG: triglyceride
TC: total cholesterol
HDLC: high-density lipoprotein cholesterol
LDLC: low-density lipoprotein cholesterol
GLU: glucose
LYMPHP: percentage of lymphocytes
NEUTP: percentage of neutrophils
MCHC: mean corpuscular hemoglobin concentration
HGB: hemoglobin
RBC: red blood cell count
HCT: hematocrit
WBC: white blood cell count
MCV: mean corpuscular volume
MCH: mean corpuscular hemoglobin
PLT: platelet
RDW-CV: the coefficient of variation of erythrocyte distribution width
RDW-SD: the standard deviation of red blood cell distribution width
PCT: plateletcrit
PDW: platelet distribution width
MPV: mean platelet volume
LYMPH: lymphocyte count
NEUT: neutrophil count
MONOP: the percentage of monocytes
MONO: monocyte count
BASO: basophils count
BASOP: the percentage of basophils
EO: eosinophils count
EOP: the percentage of eosinophils
