# Supplementary material for: Physiological and molecular dynamic changes during 23-day high-altitude exposure reveal novel biomarkers for acclimatization
Source: Front Physiol. 2026 Feb 18;17:1763837. doi: 10.3389/fphys.2026.1763837 (PMC12956708; doi:10.3389/fphys.2026.1763837)

**Supplementary information**

**S1 Table. The detailed content of 44 physiological parameters.**

| **Composite physiological parameters** | | **Single physiological parameters** |
| --- | --- | --- |
| 4 parameters | Circulation | P, SBP, DBP |
|  | Lung | SPO_2_ |
| 23 CBC counts | Red blood cell | HGB, RBC, HCT, MCV, MCH, MCHC,  RDW‒CV, RDW-SD |
|  | Platelet | PLT, PCT, PDW, MPV |
|  | White blood cell | WBC, LYMPHP, NEUTP, LYMPH, NEUT, MONOP, MONO, BASO, BASOP, EO, EOP |
| 17 biochemistry parameters | Liver | ALT, AST, AST/ALT, DBIL, IBIL, TBIL,  TP, ALB, GLB |
|  | Kidney | CREA, UA, BUN |
|  | Metabolization | TG, TC, HDLC, LDLC, GLU |

*Note: P pulse rate, SBP systolic blood pressure, DBP diastolic blood pressure, SpO_2_ saturation of peripheral oxygen, AST aspartate aminotransferase, ALT alanine aminotransferase, HGB hemoglobin, RBC red blood cell count, HCT hematocrit, MCV mean corpuscular volume, MCH mean corpuscular hemoglobin, MCHC mean corpuscular hemoglobin concentration, RDW‒CV coefficient of variation of erythrocyte distribution width, RDW-SD the standard deviation of red blood cell distribution width, PLT platelet count, PCT platelet count, PDW platelet distribution width, MPV mean platelet volume, WBC white blood cell count, LYMPHP percentage of lymphocytes, NEUTP percentage of neutrophils, LYMPH lymphocyte count, NEUT neutrophil count, MONOP percentage of monocytes, MONO monocyte count, BASO basophil count, BASOP percentage of basophils, EO eosinophil count, EOP percentage of eosinophils, TBIL total bilirubin, DBIL direct bilirubin, IBIL indirect bilirubin, TP total protein, ALB albumin, GLB globulin, CREA creatinine, UA uric acid, BUN blood urea nitrogen, TG triglyceride, TC total cholesterol, HDLC high-density lipoprotein cholesterol, LDLC low-density lipoprotein cholesterol, GLU glucose.*

**S1 Fig. Distribution and grouping of gender, eAMS status, DAHA and DAHA groups.**

a&b. Donut plots showing the counts proportion of participants by sex and eAMS status.

c. Histogram of the participants counts distribution according to DAHA.

d. Histogram of the participants counts distribution according to DAHA groups.

*Note: eAMS: experienced or experienced acute mountain sickness, DAHA: the duration of acclimatization at high altitude.*

**S2 Fig. Transcriptomic analysis of DAHA: participant distribution, gene clusters, and functional enrichment**

a. Histogram showing the distribution of the 48 participants included in the transcriptomic analysis by DAHA.

b. Histogram showing the number of participants involved in the transcriptomic analysis across four DAHA groups.

c. Heatmap illustrates the expression trends of genes across the 12 clusters over time. Red and blue colors indicate higher and lower gene expression levels, respectively, within DAHA groups.

d. Bar plot showing the enrichment analysis results of the Cluster 4 (C4).

e. Bar plot showing the enrichment analysis results of the Cluster 9 (C9).

**S3 Fig. WGCNA results of the transcriptomic data.**

a. Correlations between the soft threshold (power) and scale-free topology model fit. Red line indicates the scale-free topology fit index R^2^ = 0.9.

b. Correlation between the soft threshold (power) and mean connectivity.

c. Gene dendrogram and module assignment based on hierarchical clustering and dynamic tree cutting. Modules are identified via the soft threshold and correlations between genes, with each module represented by a distinct color.

d. Heatmap showing the Pearson correlation between each module and eAMS, as well as DAHA. The color of tiles indicates the direction of the correlation (red: positive, blue: negative), and the depth indicates the strength of the correlation. The values outside the brackets represent the correlation coefficients, whereas the values within the brackets denote the corresponding p values.

**S4 Fig. Sample clustering and enrichment analysis of DO, DM and BL**

a. Sample clustering tree before and after outlier removal.

b. Bar plot showing the enrichment analysis results of the darkorange module (DO).

c. Bar plot showing the enrichment analysis results of the darkmagenta module (DM).

d. Bar plot showing the enrichment analysis results of the blue module (BL).

**S5 Fig. The enrichment analysis results of intersection genes between modules and clusters.**

a. Bar plot showing the enrichment analysis results of the intersection genes between C4 and DM.

b. PPI results from STRING of the intersection genes between C4 and DM.

c. Bar plot showing the enrichment analysis results of the intersection genes between C4 and BL.

d. Bar plot showing the enrichment analysis results of the intersection genes between C9 and BL.

**S6 Fig. Heatmap of correlations between hub genes in Pattern 1 and Pattern 2 and detection traits.**

Light tiles represent a Pearson correlation coefficient from -1 to 0 (indicating a negative correlation), whereas dark tiles represent a correlation coefficient from 0 to 1 (indicating a positive correlation). Statistical significance is denoted as follows: * *p*<0.05, ** *p*<0.01, *** *p*<0.005.

*Note: TBIL total bilirubin, DBIL direct bilirubin, IBIL indirect bilirubin, TP total protein, ALB albumin, GLB globulin, AST aspartate aminotransferase, ALT alanine aminotransferase, CREA creatinine, UA uric acid, BUN blood urea nitrogen, TG triglyceride, TC total cholesterol, HDLC high-density lipoprotein cholesterol, LDLC low-density lipoprotein cholesterol, GLU glucose, LYMPHP percentage of lymphocytes, NEUTP percentage of neutrophils, MCHC mean corpuscular hemoglobin concentration, HGB hemoglobin, RBC red blood cell count, HCT hematocrit, WBC white blood cell count, MCV mean corpuscular volume, MCH mean corpuscular hemoglobin, PLT platelet count, RDW‒CV coefficient of variation of erythrocyte distribution width, RDW-SD the standard deviation of red blood cell distribution width, PCT platelet count, PDW platelet distribution width, MPV mean platelet volume, LYMPH lymphocyte count, NEUT neutrophil count, MONOP percentage of monocytes, MONO monocyte count, BASO basophil count, BASOP percentage of basophils, EO eosinophil count, EOP percentage of eosinophils.*

**S7 Fig. Enrichment analysis results of DEGs between eAMS+ group and eAMS- group**

a. Bar plot showing the enrichment analysis results of the up-regulated genes.

b. Bar plot showing the enrichment analysis results of the down-regulated genes.

**S1 Fig.**


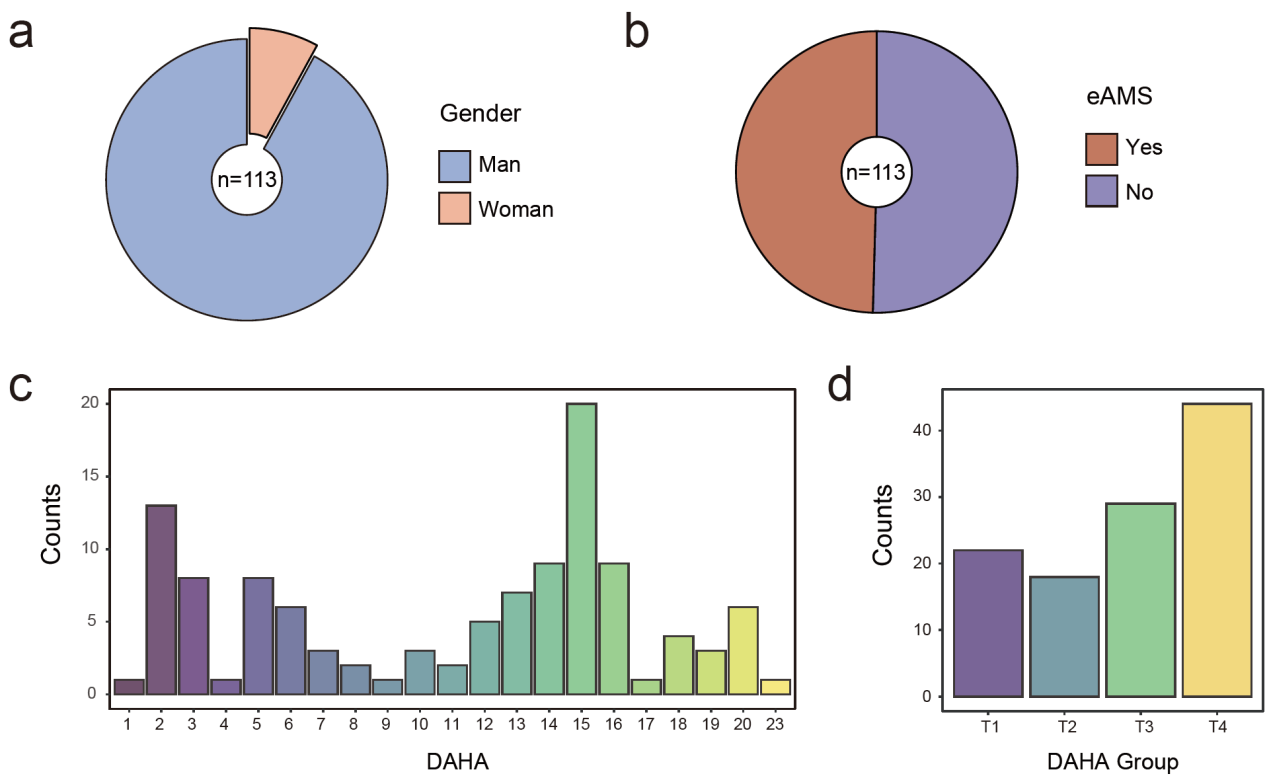


**S2 Fig.**


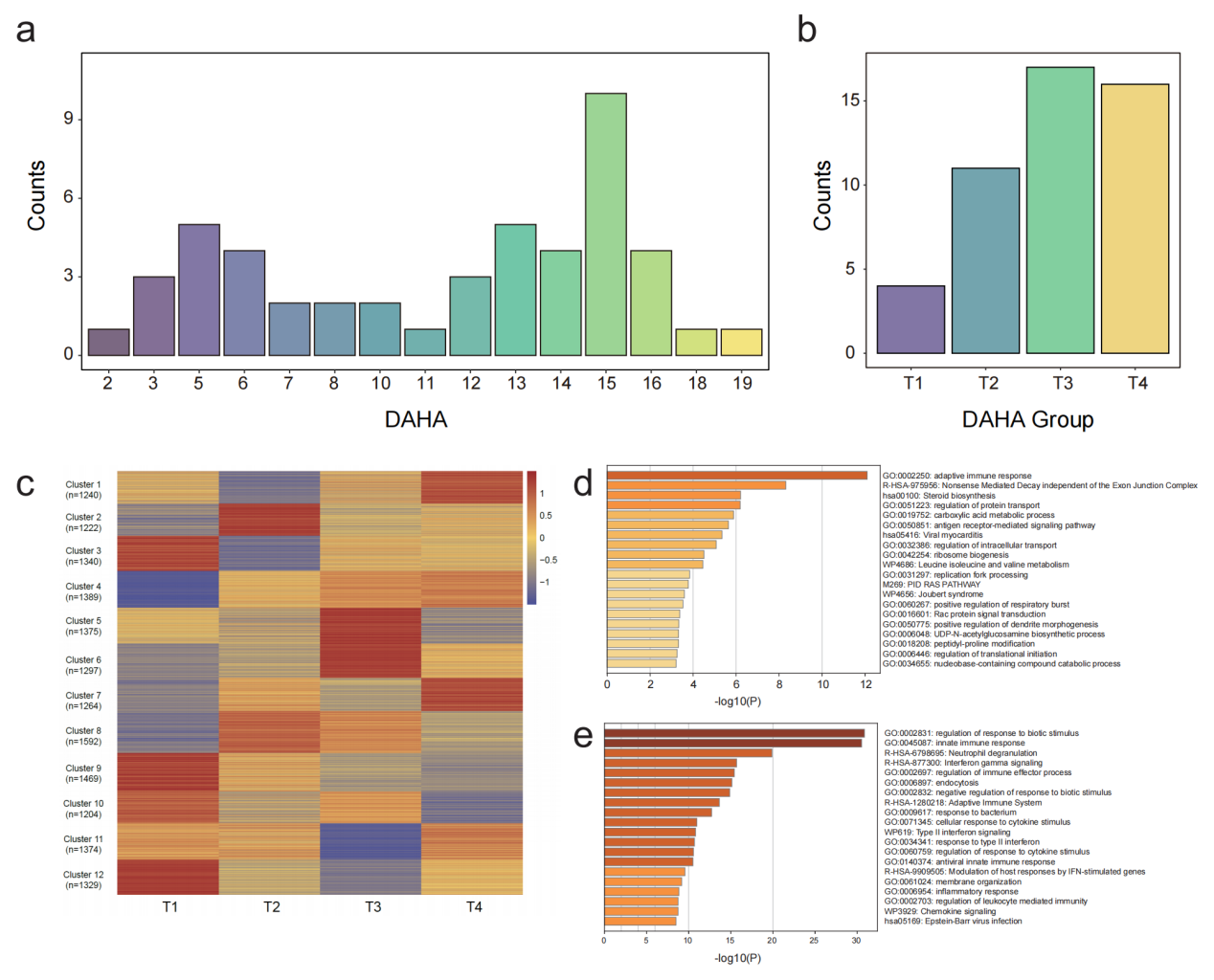


**S3 Fig.**


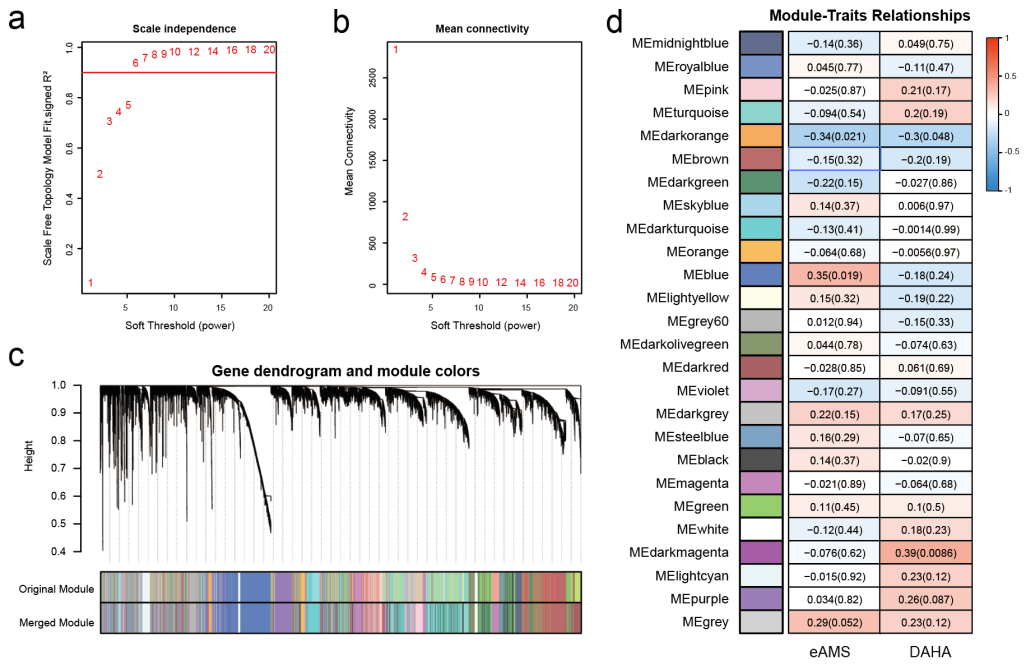


**S4 Fig.**


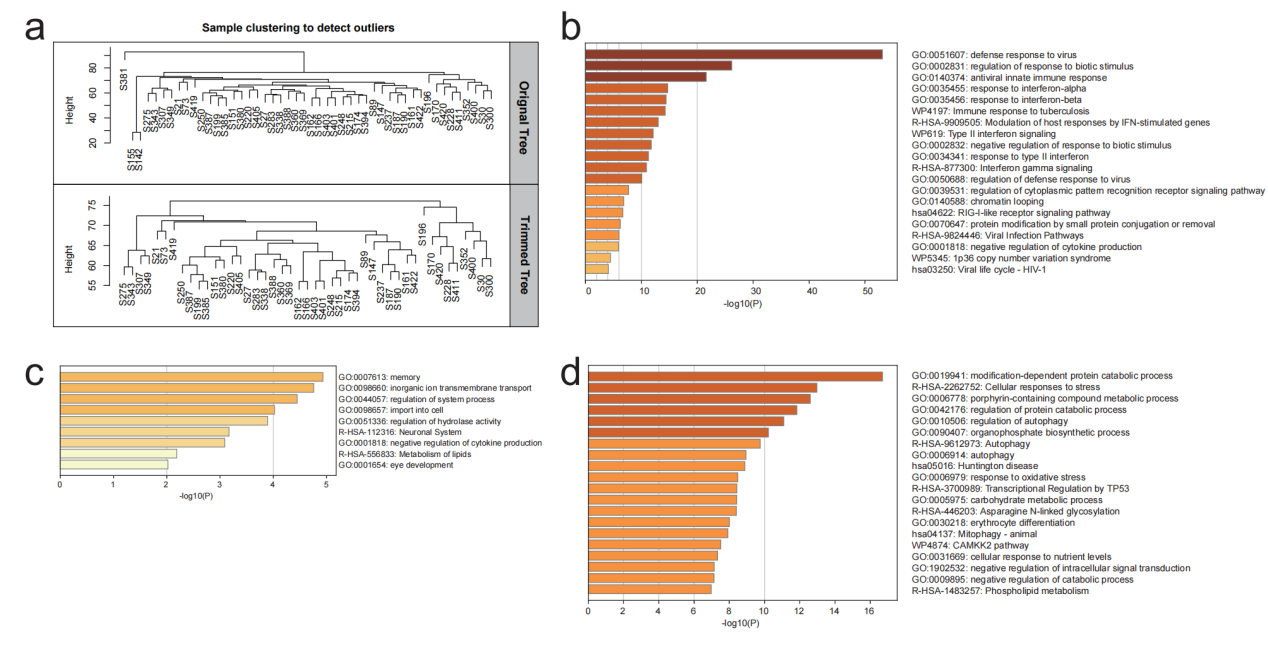


**S5 Fig.**


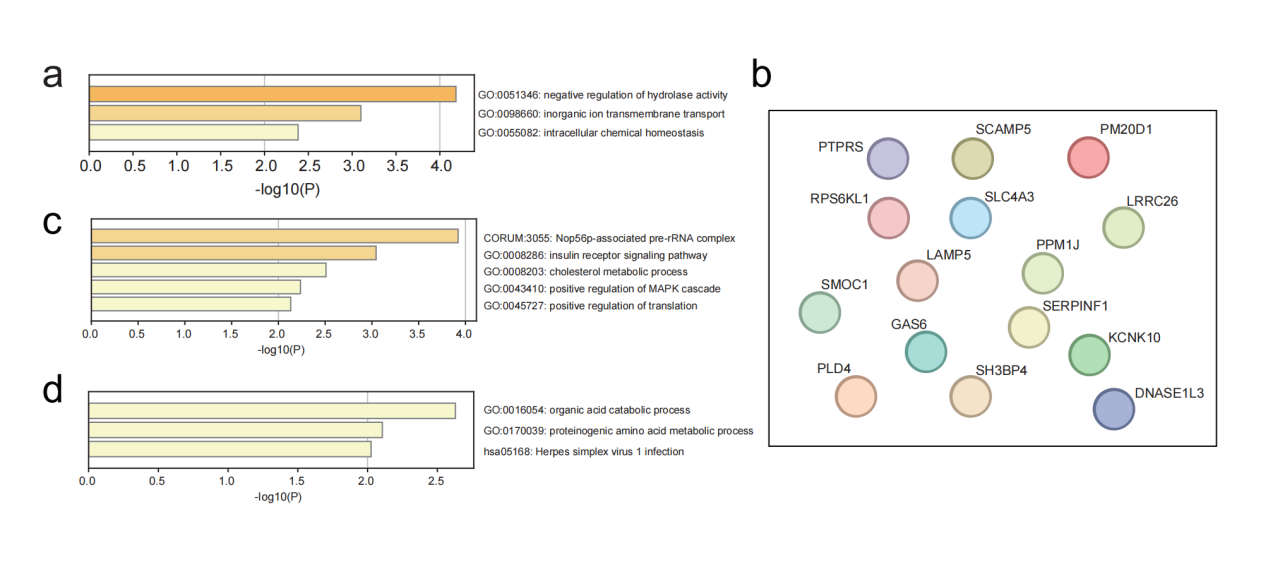


**S6 Fig.**


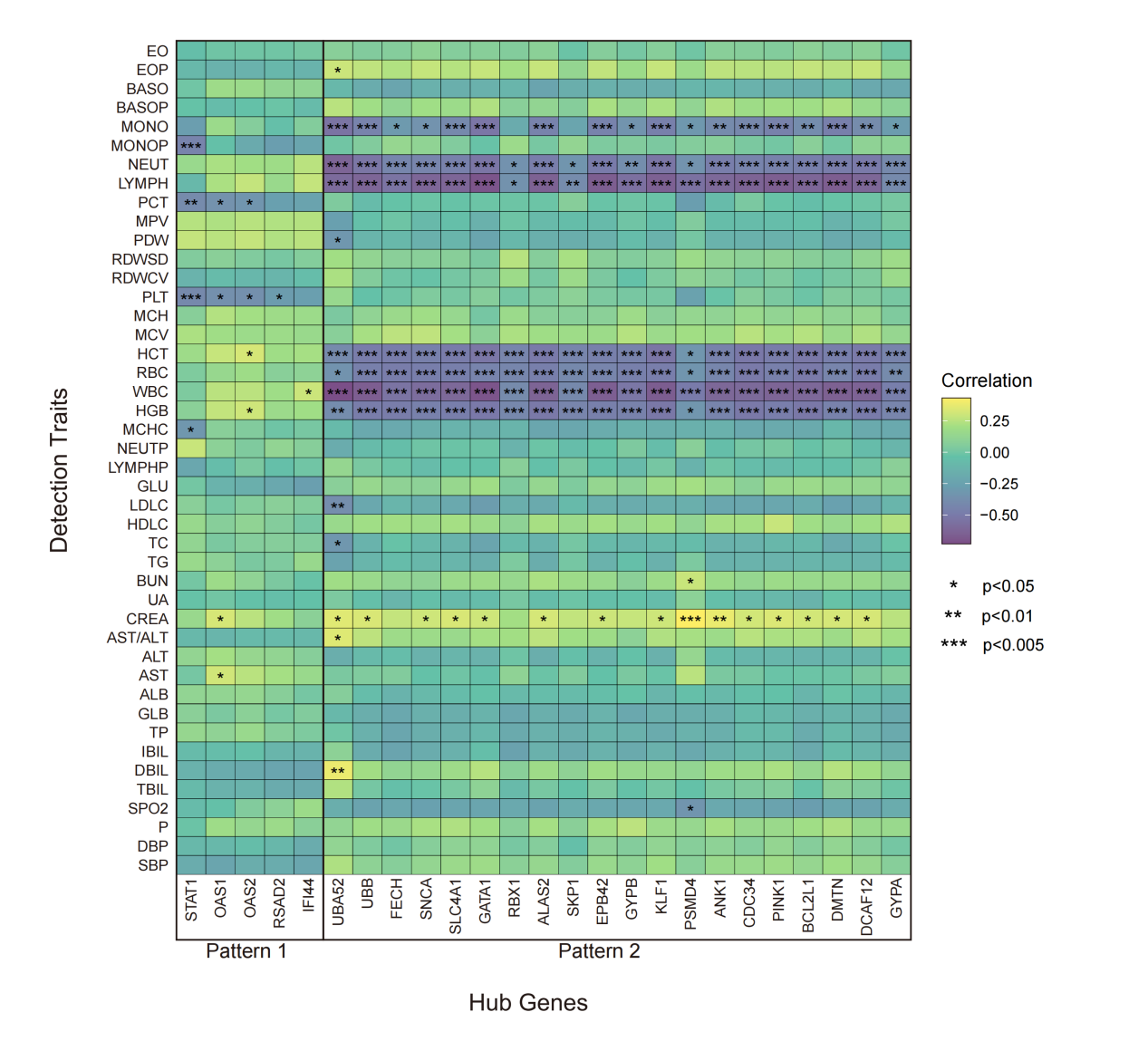


**S7 Fig.**


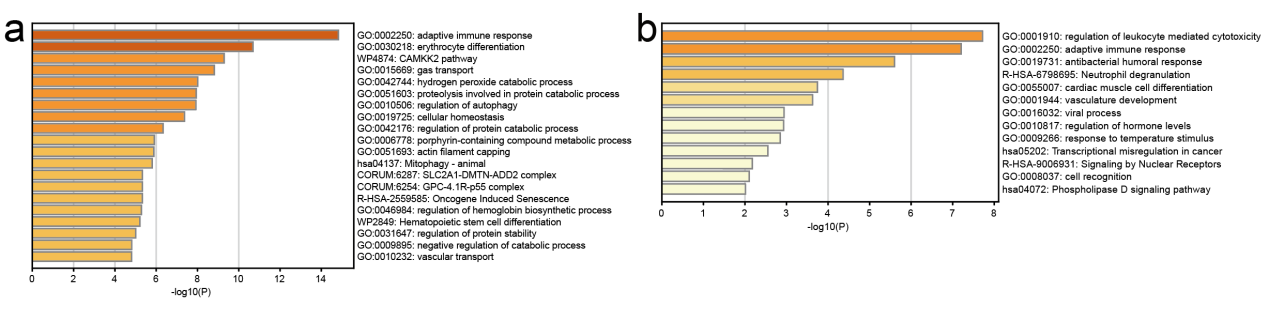

Supplement: Supplementary file 1 [file Supplementaryfile1.docx]
